# First Pediatric Case of Autoimmune Encephalitis Associated With COVID-19 in Costa Rica

**DOI:** 10.7759/cureus.30616

**Published:** 2022-10-23

**Authors:** Mariela Scheuermeier, Karina Quirós Chaves, Daniela Marín-Sanabria, Heidy Acosta-Lazo, Adriana Ulate-Campos

**Affiliations:** 1 General Medicine, Universidad de Costa Rica (UCR), San José, CRI; 2 General Medicine, Universidad de Ciencias Medicas (UCIMED), San José, CRI; 3 Pediatrics, Hospital Nacional de Niños "Dr. Carlos Sáenz Herrera" Caja Costarricense del Seguro Social (CCSS), San José, CRI; 4 Pediatric Neurology, Hospital Nacional de Niños "Dr. Carlos Sáenz Herrera" Caja Costarricense del Seguro Social (CCSS), San José, CRI

**Keywords:** refractory epilepsy, pediatric, sars-cov-2, encephalitis, autoimmune

## Abstract

Very few COVID-19-associated autoimmune encephalitis cases have been documented in children. This case report focuses on a previously healthy four-year-old girl who presented to the emergency room of the National Children's Hospital in Costa Rica in a postictal state due to a tonic-clonic seizure that progressed to status epilepticus. She had no previous history of fever or associated trauma. She was considered severe acute respiratory syndrome coronavirus 2 (SARS-CoV-2) positive by epidemiological linkage four weeks prior to the event, and her immunoglobulin G (IgG) levels for SARS-CoV-2 were positive. She presented with generalized decrease in muscle strength, she couldn’t even walk, also dyskinetic movements in upper extremities, language impairment, frequent seizures, retrograde amnesia, and orolingual dyskinesias. An extensive diagnostic workup was performed, including bacterial and viral panel in cerebrospinal fluid, however the only positive result was the IgG for SARS-CoV-2. Electroencephalogram (EEG) and magnetic resonance imaging (MRI) findings were compatible with autoimmune encephalitis.

An antibody panel was performed, which was negative in cerebrospinal fluid and positive for anti-gamma-aminobutyric acid (GABA)/b1 in serum. She received three antiseizure drugs, plasmapheresis, intravenous gamma-globulin, methylprednisolone, and rituximab, which partially improved her condition. She currently has refractory epilepsy, memory problems, loss of language skills, and neuropsychiatric dysfunction. To our knowledge, this is the first case of autoimmune encephalitis secondary to SARS-COV-2 infection in a pediatric patient in Costa Rica.

## Introduction

Encephalitis, a disease consisting of inflammation of the brain, may be caused by an infectious pathogen or by autoimmune processes [1]. Autoimmune encephalitis is a neuroinflammatory disorder characterized by diverse neuropsychiatric symptoms and autoantibodies targeting neuronal antigens [2]. These antibody targets impact synaptic transmission, causing neurophysiological dysfunction and inflammatory changes [3]. The most common autoantibodies identified in children target the N-methyl-D-aspartate receptor (NMDAR), myelin oligodendrocyte glycoprotein (MOG), glutamic acid decarboxylase 65 (GAD65), and gamma-aminobutyric acid (GABAA) receptor. It is also recognized that not all children with a clinical phenotype of autoimmune encephalitis have a known autoantibody [4].

Typically, children with autoimmune encephalitis are previously healthy and present with rapid onset of neuropsychiatric symptoms. Prodromal symptoms including fever occur in over 50% of patients [4]. Patients may present with a variety of movement disorders, they also tend to present seizures, which are the most common symptom and different types may be seen [5]. Both symptoms can be highly refractory to standard treatments. Behavioral changes are also common in pediatric autoimmune encephalitis [4].

A diagnosis should be considered in children who present with acute or subacute onset of new focal or diffuse neurologic deficits, cognitive difficulties, developmental regression, movement abnormalities, psychiatric symptoms, and/or seizures. These patients should undergo several tests and the diagnosis should not rely solely on antibody testing, as some cases may be seronegative [4]. The early initiation of primary immunosuppressive strategies is usually associated with favorable treatment outcomes [6]. Patients who fail to improve after 10-14 days should receive second-line therapies such as rituximab or cyclophosphamide, or both [5]. Outcomes range from full recovery, limited recovery in one-third of patients, to partial recovery in another third of patients [3].

The manifestations and complications of severe acute respiratory syndrome coronavirus 2 (SARS-CoV-2) infection in the pediatric population remain a developing area of investigation as the pandemic continues [3]. Compared to adults, fewer cases of coronavirus disease 2019 (COVID-19) have been reported in the pediatric population, and neurological manifestations are usually limited to headaches and dizziness. However, there are some reports of more severe manifestations such as seizures, cerebrovascular events, Guillain-Barre syndrome, myositis, and encephalitis, as presented in our case [6]. Patients in which autoantibodies have been identified are rarer and are mostly isolated case reports or case series providing limited information about this disease. In most cases with severe COVID-19 and neurological involvement, no evidence of SARS-CoV-2 detection in cerebrospinal fluid (CSF) was reported. Neurological complications of COVID-19 may occur as a result of direct virus injury, para- or post-infectious immune-mediated or inflammatory reactions triggered by SARS-CoV-2, or secondary to the systemic inflammatory response syndrome [7,8].

In this case report, we present a previously healthy four-year-old girl who presented with neurological deficits four weeks after an asymptomatic COVID-19 infection. Her clinical presentation and neuroimaging findings were compatible with autoimmune encephalitis and other alternative causes were excluded. Treatment was initiated without delay. However, the patient´s outcome was unfavorable in the end.

## Case presentation

A previously healthy four-year-old girl presented with five days of right facial pain that progressed to a global headache, associated with unquantified febrile sensation and a normal neurologic examination at the time. She had no history of fever, trauma, or other infections on previous days and she wasn’t yet vaccinated against SARS-CoV-2. Three days later she presented to the National Children’s Hospital "Dr. Carlos Sáez Herrera" in a postictal state after three generalized tonic-clonic seizures associated with episodic oxygen desaturation. The only noteworthy aspect of her medical history was that four weeks prior to this event, both of her parents had tested positive for SARS-CoV-2, so she was also considered positive by epidemiologic linkage. While in the emergency room she developed status epilepticus, requiring benzodiazepines as primary treatment. 

**Figure 1:**
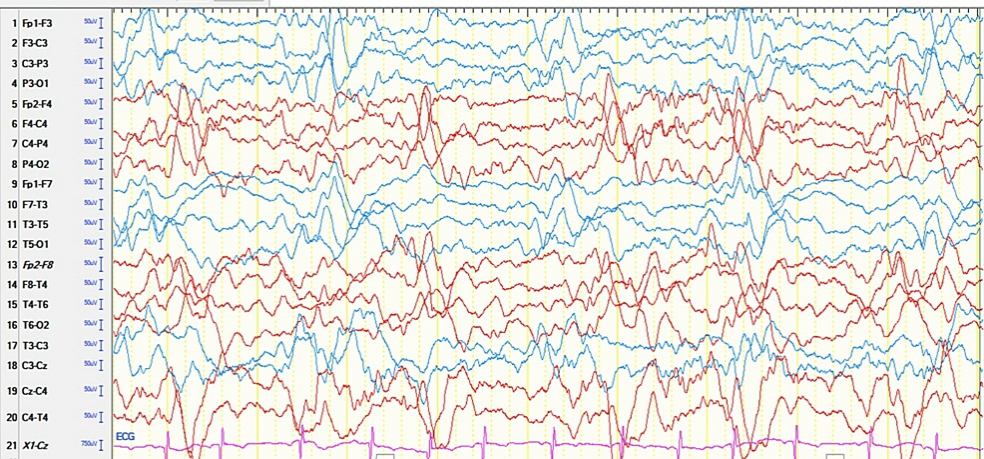
Electroencephalogram on admission. Poorly structured activity with the presence of generalized slow-wave bursts, as well as acute spike-and-waves at bilateral fronto-central-parieto-temporal areas, asynchronous, in a sub continuous manner.

Initial tests included a central nervous system computed tomography (CT), a complete blood count, and viral serologies; all of which showed no pathological findings. A nasopharyngeal swab polymerase chain reaction (PCR) was also performed, which tested positive for SARS-CoV-2. In addition, immunoglobulin (Ig) G (IgG) levels for SARS-CoV-2 were quantified, which were also positive, though IgM was negative. A lumbar puncture (LP) was performed, and the CSF analysis was normal for protein, and glucose, no red cells or oligoclonal bands were observed, and the total white blood cell count was 3 per mm3. Both culture and BioFire FilmArray (bioMérieux, Marcy-l'Étoile, France) in CSF samples were negative. The initial electroencephalogram (EEG) showed poorly structured activity with generalized slow wave bursts, as well as asynchronous, sub-continuous spikes and waves at bilateral fronto-central-parieto-temporal areas (Figure 1).

Based on the clinical findings, and after ruling out other possible causes, the etiology of the patient's condition was considered to be a post-infectious reaction related to COVID-19. She was admitted for complementary studies and, since the patient met several criteria described by Graus et al. for autoimmune encephalitis, treatment was started with intravenous (IV) gamma globulin [9]. 

The following day she deteriorated as a result of a prolonged convulsive status epilepticus, which was not monitored on EEG, and lasted approximately 40 minutes, she then required treatment with diazepam and phenobarbital. Cognitive deterioration was observed, in addition to difficulty breathing and oxygen desaturation. Consequently, the patient was intubated and transferred to the pediatric intensive care unit (PICU). She required mechanical ventilation and continuous treatment with phenytoin and methylprednisolone. Three days later, she was extubated due to clinical improvement. Thereafter, she associated clinical features such as language disturbances, retrograde amnesia, motor disturbances, sleep disturbances, choreoathetotic movements in the upper extremities, and orolingual dyskinesia. She continued to experience approximately eight to 10 seizures daily, so treatment with phenobarbital was initiated.

**Figure 2:**
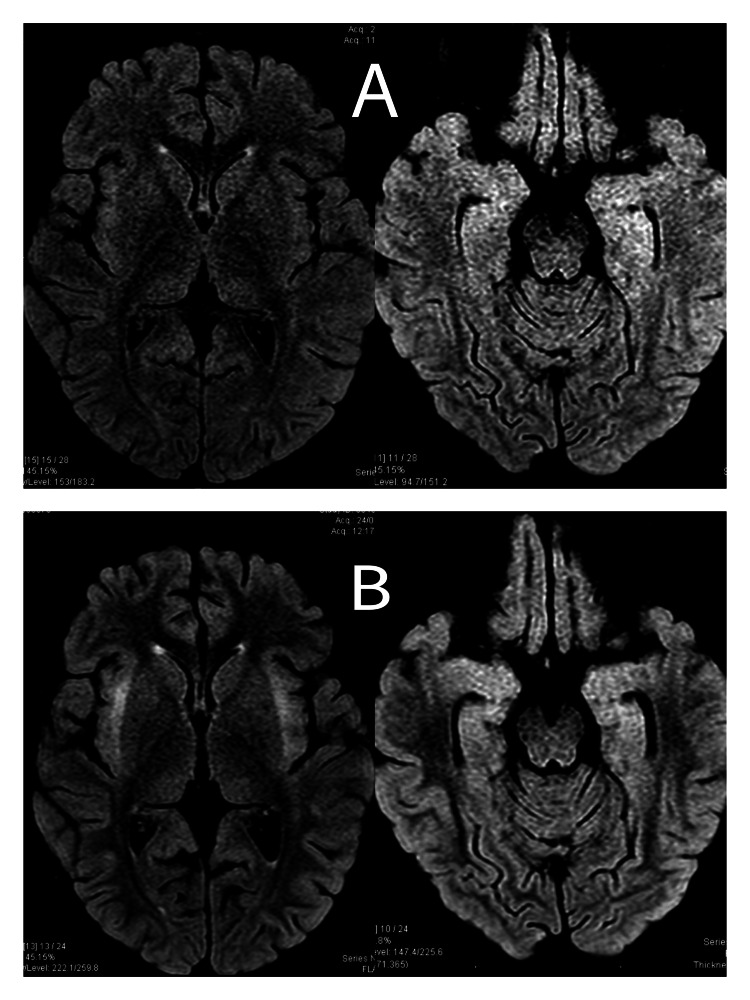
FLAIR MRI sequences prior (A) and after (B) IV steroid treatment. A) MRI prior to the start of IV steroid treatment: Left shows discrete diffuse volume loss is observed. In addition, right shows hyperintensity in both temporal poles and at the right insular and left parietal level (right). B) MRI after completing 10 days of treatment with IV steroids. Left shows insular, external capsule, and claustrum signal change is observed, and the volume loss appears to be increased after the steroid treatment. Right shows evidence of an increased signal in both insulae and medial temporal lobes. In addition, it has no enhancement with gadolinium and does not restrict diffusion sequences. FLAIR: fluid-attenuated inversion recovery

Two MRIs were performed, as shown in Figure 2, which revealed a loss of volume probably related to steroid treatment as well as hyperintensities both in T2 and fluid-attenuated inversion recovery (FLAIR) sequences in the bilateral insular cortex and medial temporal region, topography also compatible with autoimmune encephalitis. Seven days after symptom onset, nasopharyngeal swab PCR for SARS-CoV-2 was repeated and it turned out negative. In addition, a serum antibody panel (including anti-NMDAR, anti-CASPR2, anti-AMPAR, anti-LG1, anti-DPPX and anti-GABA/B1) was positive for anti-GABA/B1, but the same antibody was negative in CSF.

Despite antiseizure drugs, she still presented numerous short seizures daily, so vigabatrin was initiated. She was also started on plasmapheresis, however, only three out of five cycles could be completed due to venous access complications. Nevertheless, clinical improvement was noted. 

The number of daily seizures decreased, dyskinetic movements were almost imperceptible, motor skills improved, language was more fluent, yet still repetitive, and her retrograde amnesia began to diminish slowly. Since treatment with plasmapheresis could not be completed, it was decided to initiate rituximab as an immunomodulatory treatment.

During the remaining time of hospitalization, the patient persisted with two or three self-limited focal daily seizures, characterized by left gaze deviation, with complete resolution within a few minutes. 

Clinically, other functions such as walking, eating, sphincter control, and fine motor skills were almost restored. In addition, speech was more organized, less repetitive and sleep patterns were normal. Due to clinical improvement she was discharged a few days later, maintaining treatment with phenobarbital, vigabatrin and carbamazepine. 

A month later, the patient was admitted again to start treatment with rituximab at a dosage of 285 mg IV. She completed a four-cycle regimen with this dose, with no reported adverse effects. Despite completing treatment with rituximab and having optimized antiseizure therapy, the patient had decreased language comprehension and production. The number of daily seizures increased, which were mostly characterized by focal-onset motor seizures without loss of consciousness. In addition, atypical new behaviors were recorded, such as wall licking, hyperactive behavior, self-limited violent behavior, periods of inattention, and inability to interact with other children. Following these new clinical findings, it was decided to discontinue treatment with phenobarbital, as it can worsen behavioral disorders, and carbamazepine was added instead. Due to her speech impairment, otoacoustic emissions, automated auditory evoked potentials, and tympanometry were performed; which ruled out any recent-onset hearing deficits that could potentially explain this speech deficit. Currently, the patient experiences two to three seizures daily, with the same characteristics described above, however, a small improvement was noted with the addition of carbamazepine. Her current antiseizure medication also includes vigabatrin and valproate. Her language deficit remains. She currently produces no words, and the behavioral changes described above persist. 

## Discussion

A thorough search was performed and very few cases reported in the literature on encephalitis secondary to COVID-19 in children were found. We will compare three that had key similarities to our case. 

The first case was a 23-month-old patient who presented with sleep disturbances, hyporexia, and constipation. During hospitalization, she developed hyperkinetic limb movements and seizures, much like our patient. She was positive for SARS-CoV-2 on nasal swab PCR and IgG, but CSF was also negative. Treatment with steroids and intravenous gamma globulin was also provided in this case, leading to significant clinical improvement. Anti-NMDA receptor antibodies were isolated in CSF, classifying it as COVID-19-associated anti-NMDA receptor encephalitis [10].

The second case was a seven-year-old boy who developed acute ataxia that progressed to encephalopathy over the following days, also presenting with seizures and choreiform movements, similar to our case. He also tested positive for SARS-CoV-2 on throat swab reverse transcription PCR (rtPCR). The child had lymphopenia so the initial treatment consisted of antibiotics, acyclovir, and three courses of plasmapheresis. Subsequently, he received methylprednisolone in a descending schedule at a dose of 30 mg/kg/day IV for five days, followed by 20 mg/kg IV for two days, and finally prednisolone 2 mg/kg p.o. He gradually improved within two weeks and was discharged ambulating but mildly ataxic. He continued treatment with an oral prednisolone taper and antiseizure drugs [11].

The third case was a five-year-old girl who presented with cough, fever, and neck swelling, but later on developed altered mental status, irritability, sleepiness, and lethargy. The rtPCR test for SARS-CoV-2 using a nasopharyngeal swab was positive. The EEG showed a slow base rhythm with synchronous bilateral potentials formed by slow waves, similar to the findings in our patient’s case. MRI diffusion-weighted imaging (DWI) showed hyperintense focal lesions compatible with edema in the splenium of the corpus callosum and the right parietal subcortical area. CSF was within the normal range and also negative for SARS-CoV-2. After a cycle of high-dose IV methylprednisolone, a quick and complete remission of symptoms was noted. The patient was discharged two weeks later, but unlike our patient, she had no persistent sequelae after the infection [1].

Clinical manifestations of autoimmune encephalitis vary depending on the specific antibody or mechanism that causes it. The most common in pediatric population is NMDA-R-mediated encephalitis. It affects mostly females in a 4:1 ratio and is frequently associated with ovarian teratomas, although it is less common in children under 10 years of age. It usually manifests with psychotic behavior and alterations in affective features. The classic presentation includes seizures, movement disorders such as orofacial or limb dyskinesia, changes in levels of consciousness, and autonomic dysfunction [10]. Our patient presented with prolonged seizures, diskinetic movements and behavioral disorder, so clinically we suspected autoimmune encephalitis. Later on, she had clinical deterioration probably related to ongoing seizure activity.

Another antibody-mediated syndrome includes antibodies against GABA receptors, type A may be present in children or adults, while type B is much more common in adults. These patients associate limbic encephalitis and refractory epilepsy [12].

Antibodies are frequently not isolated in CSF, and the etiology is suspected based on the clinical picture. This may occur for several reasons: low titers or methodological problems may result in false negatives, there could be a mismatch with clinical features, the antibodies themselves may be unknown, and, in some cases, mechanisms not associated with antibody-mediated immunity could be responsible [2]. In our case, we isolated a GABA-B receptor antibody in the serum panel, but the clinical characteristics are more compatible with anti-NMDA receptor encephalitis, but the CSF panel was negative. It is worth mentioning that serum neuronal antibodies have been identified in 6.4% of children presenting with seizures under three years of age, none of which had encephalitis, and the most frequent was GABA-B receptor [13]. Our patient presented with seizures, she is four years old, but this fact must be taken into consideration as serum antibody positivity in children presenting with seizures is usually non-specific. The clinical features and evolution that our patient presented are compatible with autoimmune encephalitis, but we believe that serum GABA-B receptor antibodies were a false positive. Isolation of SARS-CoV-2 in CSF can be difficult because of its transient dissemination and the fact that its CSF titer may be extremely low, as seen in our case and in the literature [13].

## Conclusions

Autoimmune encephalitis can occur as a postinfectious sequel of COVID-19 in children, even if they did not develop any severe symptoms in the acute infection. In these cases, it is uncommon for the virus to be isolated in CSF, however, this fact does not rule out the diagnosis. 

The most relevant and consistent clinical presentation appears to be seizures, which are usually refractory to treatment, in addition to various alterations of higher-order mental function such as language and memory deficits, abnormal movements, and sleep disturbances; as witnessed in our patient’s case. She had positive serum anti-GABA-B antibodies, however, they were negative in CFS, so we considered this a false positive. The only infectious parameter found in her was a positive PCR for SARS-CoV-2, positive IgG levels for SARS-CoV-2, and negative IgM, therefore, we conclude that this is the first pediatric case of autoimmune encephalitis secondary to COVID-19 in Costa Rica.
